# Beyond physiology: Acute effects of side-alternating whole-body vibration on well-being, flexibility, balance, and cognition using a light and portable platform A randomized controlled trial

**DOI:** 10.3389/fspor.2023.1090119

**Published:** 2023-01-30

**Authors:** Yannik Faes, Cornelia Rolli Salathé, Marina Luna Herlig, Achim Elfering

**Affiliations:** ^1^ Business Psychology, Lucerne University of Applied Sciences and Arts, Lucerne, Switzerland; ^2^ Faculty of Psychology, Distance University, Brig, Switzerland; ^3^ Department of Work and Organizational Psychology, University of Bern, Bern, Switzerland; ^4^ Department of Psychology, University of Fribourg, Fribourg, Switzerland

**Keywords:** whole-body vibration (WBV), musculoskeletal, flexibility, balance, cognition, inhibition, stroop-color-word interference task, SLIP trip and fall accidents

## Abstract

A good body-balance helps to prevent slips, trips and falls. New body-balance interventions must be explored, because effective methods to implement daily training are sparse. The purpose of the current study was to investigate acute effects of side-alternating whole-body vibration (SS-WBV) training on musculoskeletal well-being, flexibility, body balance, and cognition. In this randomized controlled trial, participants were randomly allocated into a verum (8.5 Hz, SS-WBV, *N* = 28) or sham (6 Hz, SS-WBV, *N* = 27) condition. The training consisted of three SS-WBV series that lasted one-minute each with two one-minute breaks in between. During the SS-WBV series, participants stood in the middle of the platform with slightly bent knees. During the breaks in between, participants could loosen up. Flexibility (modified fingertip-to-floor method), balance (modified Star Excursion Balance Test), and cognitive interference (Stroop Color Word Test) were tested before and after the exercise. Also, musculoskeletal well-being, muscle relaxation, sense of flexibility, sense of balance, and surefootedness were assessed in a questionnaire before and after the exercise. Musculoskeletal well-being was significantly increased only after verum. Also, muscle relaxation was significantly higher only after verum. The Flexibility-Test showed significant improvement after both conditions. Accordingly, sense of flexibility was significantly increased after both conditions. The Balance-Test showed significant improvement after verum, and after sham. Accordingly, increased sense of balance was significant after both conditions. However, surefootedness was significantly higher only after verum. The Stroop-Test showed significant improvement only after verum. The current study shows that one SS-WBV training session increases musculoskeletal well-being, flexibility, body balance and cognition. The abundance of improvements on a light and portable platform has great influence on the practicability of training in daily life, aiming to prevent slip trips and falls at work.

## Introduction

Slips, trips and falls (STF) are the most frequent accidents in Switzerland, causing 27.7% of all accidents, i.e., about 70,000 workers, in 2020 (1). Also, STF are the most expensive accidents and yielded 41% of all accident expenditure for the years 2014 to 2018 combined. In order to be able to reduce STF incidents, risk factors must be identified (2).

One important risk factor of falls is a weak balance (3), which is related to muscle weakness (4). However, to predict fallers is difficult, because there are several risk factors, which include motor, sensory, and cognitive processes (3). Due to loss of balance being a possible influence of individual frailties on STF (5), balance trainings are recommended to reduce STF (6, 7). Most tested balance trainings often are time-consuming, need much advise, put other regulatory efforts like change of clothes or place which increase regulatory demands at work and therefore low compliance and drop out often occurs (8, 9). The training goal with respect to prevention is that the training is short and easy to administer but also easy to adjust to individual condition and there is no need for change of clothes, shoes, or location (10). In addition, there should be a benefit from each single training session that is noticeable. Thereby, it would be an advantage, when the benefit of a single training is not only improved body balance but includes other improvements like improved mental functions as well. Multiple benefits make it more likely that the training is accepted and becomes a routine behaviour. Also, to increase long-term adherence, it is important to build a routine around a person's lifestyle (11).

In the current study, whole-body vibration (WBV) training is introduced as an exercise-based health-intervention to improve balance with the aim to reduce STF not only in terms of improving motor and sensory, but also cognitive processes.

### Healthy and unhealthy forms of whole-body vibration

Long lasting vibrations are biomechanical risk factors that contribute to the development of musculoskeletal pain (12). Other mentioned biomechanical risk factors are heavy load lifting, bending and twisting and remaining in a static position over longer time (13). Vibration exposure from driving vehicles or from vibrating, hammering or rotating work equipment may lead to musculoskeletal and neurological disorders, depending on strength, frequency, duration of action, working method and body posture (14, 15). For example, vibration experienced by construction workers handling compressed air hammers or truck drivers during long-term journeys can cause vascular, neurological and musculoskeletal problems, as well as disturbances of the lumbar spine and the associated nervous system (16).

However, a large number of studies have shown that shorter exposure on vibration can also have a prophylactic effect on musculoskeletal discomfort when range of vibration frequency, amplitude and duration are properly dosed. Thus, in addition to a reduction of musculoskeletal disorders, WBV training can also promote improvements in sensorimotor and muscular performance, balance, functional mobility, bone mineral density, maximum and rapid force, stretch reflexes, and speed of movement (17–25).

WBV exercises are easily applied (10). As the exercise is not exhausting, users usually do not sweat during training sessions. WBV is easily adapted to individual level of body balance and fitness. Hence, users do not have to change clothes or shoes, or take a shower afterwards, which could be important in occupational settings or in healthy young adults, where users do not want to waste time on an intense worksite activity training. High training durations often result in a lack of participation and compliance rate (8, 9). According to worksite training studies, the duration of one WBV training session is about 10 min (26, 27), which is half the time participants usually have to invest in worksite activity trainings (28).

After brief instructions concerning the correct body posture and the handling of the vibration platform, participants can start WBV exercises, which have proven to gain high compliance rates (26, 29). A three-month WBV-intervention with employees suffering from chronic low-back pain, revealed a compliance rate of 81.1%, with two to three recommended trainings per week (29). A four-week WBV-intervention study with office-workers of a Swiss federal department even revealed a training attendance of 129%, therefore more than the instructed three trainings per week (26).

### Different forms of whole-body vibration training

In their systematic review, Oliveira and colleagues (30) found adverse events in only 55 of 1,833 volunteers, who mentioned experiencing for example back pain, pain in their legs, or dizzy sensations. With only 3% adverse events, WBV training is therefore considered to be relatively safe (30). Rogan and colleagues (31) have differed three types of WBV. Sinusoidal vertical WBV (SV-WBV) and sinusoidal side-alternating WBV (SS-WBV), which use a single vibrating platform, and stochastic resonance WBV (SR-WBV), which functions with two independent powered platforms, which can be comparable to skis.

Vibration frequencies among sinusoidal WBV are constant, whereas SR-WBV works with unpredictable random frequencies forcing the human body to constantly adapt its neural and muscular reactions (32). To the best of our knowledge, SR-WBV was originally developed to increase performance of professional ski athletes. Nowadays it is used in different sports as injury prevention, but also in therapies with Parkinson patients (33), stroke patients (34), frail elderly (32), or patients with chronic low back pain (35). However, devices working with SR-WBV are big, heavy and expensive and thus rather used in physiotherapy or in fitness centers than at home.

**Figure 1:**
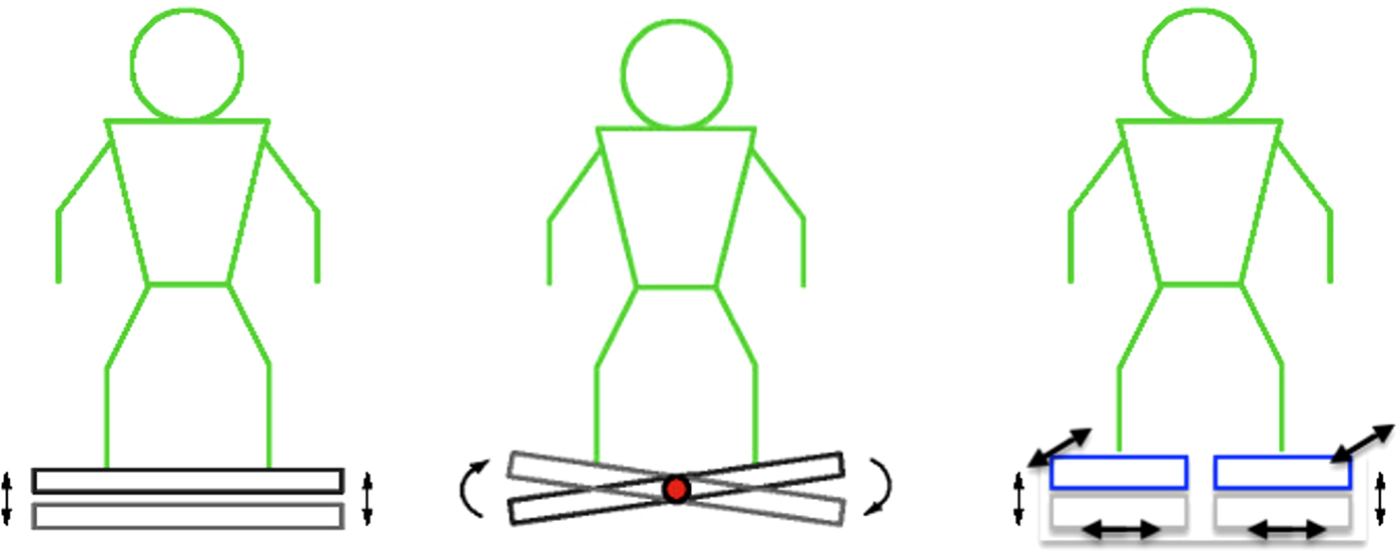
Different types of vibrating platforms. Whole-body vibration (WBV) types from left to right: sinusoidal vertical (SV-WBV), sinusoidal side-alternating (SS-WBV), and stochastic resonance (SR-WBV); source: mediplate.ch; SR-WBV customized by the author.

In the current study, SS-WBV is applied by using a light and portable platform, which seems to be ideal as a training device whether working from home or onsite. Although, SS-WBV seems to have a higher effect than other forms of WBV on bone mineral density (30), in terms of load, Rohlmann and colleagues (36) showed that the maximum load on the vertebral body was lower in SS-WBV (15%) than in SV-WBV (27%). This might be due to the fact, that in comparison to SV-WBV where both legs move up and down at the same time, in SS-WBV, the oscillations take place around a pivot at the center of the platform. Due to this, users have to alternate vibrations between both sides, i.e., when the right foot moves up, the left foot moves down, and vice versa (30, 37). The different types of vibrating platforms are illustrated in Figure 1.

WBV training has proven to be a safe and useful way to improve performance in athletes among different sports (38). However, as elite athletes already have a high level of performance, WBV training often leads to bivalent results, because it might be too unspecific to improve sport-specific strength, flexibility or balance (39, 40). Not only have recent studies revealed that WBV training is especially beneficial to improve stability or functional mobility in the elderly population (41), in patients with low-back pain (42) or in stroke patients (43), promising effects, such as increased balance and musculoskeletal well-being were also found at the workplace, e.g., in office workers who spend much time in sitting position (26). Recently, even individuals affected with COVID-19 who performed WBV training have exhibited improvement in inflammatory status and an overall improvement in quality of life. Moreover, a reduction of time in intensive care units in severely affected patients was also identified (44).

The current study aims to add knowledge on SS-WBV training effects by use of an experimental design. Experimental evidence for SS-WBV training effects is an important first step before an examination of this portable platform as training device for work from home and onsite occurs in future studies. Therefore, the focus of the current study is on acute musculoskeletal and cognitive effects of SS-WBV in laboratory, unprecedentedly using a light and portable platform with young and healthy participants. Although SS-WBV effects would be expected to be stronger in an older and unhealthy sample, effects are also expected to be meaningful in young and healthy individuals.

Another goal of the current study is to be able to observe side-effects. Health risks increase simultaneously as vibration intensity and exposure increase. However, according to Seidel et al. (1986), vibrations under 20 Hz are safe (45). In the current study, frequencies under 10 Hz are applied, thus side effects are not expected. If no side-effects are observed, future studies might include older people or frail individuals as well.

### Cognitive interference and its relations with physical parameters

Body balance depends on musculoskeletal and cognitive function. Together with attention, cognitive flexibility and decision making, inhibitory control belongs to the executive functions (46). Executive functions are located in the prefrontal cortex and are responsible for higher order cognitive abilities, e.g., volitational control over goal-directed behavior (47–50). Thus, goal-directed behavior may be volitionally achieved by deliberately suppressing dominant, automatic responses or impulsive reactions (49, 51). For example, when we walk in the park our automatic tendency would be to place one foot in front of the other. However, when suddenly facing a slippery or unstable surface, inhibitory control helps us to stop this automatic behavioral tendency, which must quickly be modified (52).

Two subcomponents of inhibitory control are motor response inhibition, i.e., the process of revoking an impulsive reaction, and cognitive interference inhibition, i.e., the ability to withstand stimuli related interference of the external environment (53, 54). The latter is subject of the Stroop Test and is subsequently referred to as “cognitive interference”.

Due to the incongruent occurrence of two stimuli (color and description) in the validated Stroop Test (55), the examinee perceives the occurrence of the stimuli as unwanted, sometimes even disturbing, which are two of the defining characteristics of cognitive interference (56). To enable the required performance, participants must ignore the written name of the color. This allows them to name the ink color of the word, which is a goal-directed behavior that requires a little more processing time. Hence, a resulting correlation with poorer performance seems obvious (56).

According to Bolton and Richardson (2022), inhibitory control has proven to be a significant and unique factor in fall prevention (52). Motor training combined with cognitive interference tasks plays an important role, especially for people with Parkinson's disease (46) or older people who participate in fall prevention training (57). These results prompted us to go a step further. Fall-safe older people are more active and safer than their peers, which in turn can lead to a change in physical well-being and not only includes physical activity and balance, but flexibility as well (58). As recently demonstrated, vibration can improve balance in older people (59), as well as in individuals with metabolic syndrome (60) preventing falls and injuries. Thus, it could be that vibration contributes to improved surefootedness. Faes et al. (2018) were able to show that WBV improved surefootedness - and that WBV has a positive effect on balance in addition to surefootedness in healthy individuals (26).

Regular training is especially important with regard to balance, which is an important part of physical well-being, daily mobility and therefore general ability to function in everyday life (61). This is consistent with improved neuromuscular control leading to a better postural stability achieved through WBV training (62). Furthermore, good postural stability has a positive influence on balance (58).

Specifically with regard to the young and healthy participants in the current study, McClain and Shallen (2015) demonstrated that WBV can improve participants fitness, thus also balance, better than static training. In another study conducted by Despina et al. (2020), WBV training resulted in superior short-term performance improvements in flexibility, strength and balance compared to an equivalent exercise without vibration (63) and thus confirmed similar results from Ritzman et al. (2014) (64). Exercise and training programs that include WBV can therefore provide additional benefits for young and well-trained adults.

The current study aims to find new insights in WBV and their effect on musculoskeletal well-being, muscle relaxation, sense of flexibility, sense of balance, and surefootedness. Specifically, based on previous research as stated above, musculoskeletal well-being, flexibility, and balance should be increased and cognitive interference decreased after one training session of SS-WBV. These results are hypothesized to be found only in the experimental group (8.5 Hz) and not in the control group (6 Hz):

Musculoskeletal well-being and muscle relaxation assessed with questionnaire is expected to be increased after one training session of SS-WBV with a vibration frequency of 8.5 Hz but not of 6 Hz (H1). Also, flexibility assessed through the modified fingertip-to-floor method (mFTF) is expected to be increased after one training session of SS-WBV with a vibration frequency of 8.5 Hz but not of 6 Hz (H2). Furthermore, balance measured with the modified Star Excursion Balance Test (mSEBT) is expected to be increased after one training session of SS-WBV with a vibration frequency of 8.5 Hz but not of 6 Hz (H3). Lastly, cognitive interference measured with the Stroop Color Word Test is expected to be decreased after one training session of SS-WBV with a vibration frequency of 8.5 Hz but not of 6 Hz (H4).

## Materials and methods

### Ethics

The study was performed in consensus with all requirements defined by the Swiss Society of Psychology and was conducted with the understanding and the consent of the human subject. The Ethical Committee of the responsible University faculty (University of Bern) has approved the study (Nr.: 2019-07-00005).

### Participants

Number of participants was calculated using G-power software. A moderate effect size was chosen as a standard in this calculation (65). The required sample size for each exercising condition – verum and sham - was 28 participants, expecting a moderate effect size (*d* = 0.5) for the t-test analysis between two dependent means and a requirement of 90% power.

Participants with one or more of the following criteria were excluded: Being pregnant, having osteosynthesis material (such as implants or screws) in the body, musculoskeletal disorders, joint problems (especially regarding the knee, hip, and back), herniated discs, rheumatism (such as spondylitis, gout, osteoporosis, osteoarthritis), cardiovascular complaints, disorders related to the sense of balance (such as hearing loss). Also, participants were advised to attend the study in a rested state and must not have had any intensive workout within the previous 24 h, because of musculoskeletal and cognitive effects. In order to attend the Stroop Test (66), participants must also not suffer from red-green color blindness or take medication known to affect the central nervous system.

A number of 55 students and acquaintances signed up for the study. No participants had to be excluded before, during or after the experiment. Body mass index was calculated as a participants weight in kilograms divided by height in meters squared. Students who participated were reimbursed with one of 15 mandatory participant-hours by the associated university. Acquaintances were thanked with sweets after the experiment.

### Vibrating Platform

Two vibration platforms named MediPlate® (Dormena GmbH, Liestal, Switzerland) were used in the current study. They reach frequencies between 6 and 13 Hz of ball-bearing side-alternating (rocking) vibrations. The MediPlate® represents a transportable vibration platform as it weighs only 15.5 kg and is rather small (length: 77 cm, width: 44 cm, height: 12.5 cm). Amplitude is between 2 mm and 8 mm depending how participants place their feet on the platform. In the current study, participants exercised with an amplitude of about 5 mm.

The verum condition was set at a frequency of 8.5 Hz (Level 20). It is experienced as slightly higher than the minimal stimulation parameter of 6 Hz (Level 1), which was used as sham condition. Acceleration forces – calculated as f(max) = amplitude * (2*π* * frequency)^2^−were 12.8 m/s^2^ (1.3 g) in the verum condition and 5.3 m/s^2^ (0.5 g) in the sham condition. Thus, forces of the verum condition on the body were lower than walking, which reaches between 2.7 g and 3.7 g (67).

Since there were no studies on the MediPlate® vibration platform so far, our decisions concerning chosen frequencies rely on experience with various vibration training studies (10) combined with recommendations from the designers of MediPlate®. A blank control group without any vibration was not carried out to ensure that participants were unaware of their group-allocation.

### Musculoskeletal well-being and muscle relaxation assessed with questionnaire

Musculoskeletal well-being and muscle relaxation were assessed with a short version of the self-administered questionnaire of Burger et al. (2012) before and after the exercise (3). The questions started with the lead-in phrase, “How do you rate your personal sensations regarding muscles and joints (back, shoulders and neck, legs) at this moment?” and were answered on a 100-point-rating-scale from zero (“not at all comfortable/relaxed”) to 100 (“as comfortable/relaxed as possible”).

### Flexibility assessed through the modified fingertip-to-floor method (mFTF)

Flexibility was assessed through the modified fingertip-to-floor method (mFTF). Compared to the original fingertip-to-floor method (FTF), where participants stand on the floor, participants stand on a box when attending the mFTF. This is an advantage as measurements of participants who are able to touch the floor or reach beyond can still be included (68). Participants are asked to bend over as far as possible keeping their legs and arms straight, while the examiners measured the distance to the box. This procedure was repeated three times. Gauvin et al. (69) reported high test-retest (*r* = 0.98), as well as high inter-rater reliability (*r *= 0.95) for the mFTF.

Additionally, sense of flexibility was assessed in one question: “How flexible do you feel at this moment?” (17). Answers could range from 0 being “a lot worse than usual”, to 100 being “much better than usual”, and with 50 being “same as always”.

### Balance measured with the modified star excursion balance test (mSEBT)

Balance was measured with the modified Star Excursion Balance Test (mSEBT) (70). In this test, dynamic balance is assessed in the eight directions anterior, anteromedial, medial, posteromedial, posterior, posterolateral, lateral und anterolateral with both high intra-test (*r* = 0.84 to 0.93) and test-retest reliability (*r* = 0.89 to 0.93) (70, 71). Excursion distances were normalized to individual leg length of each participant, in order to exclude effects related to gender, because males were found to have significantly greater excursion distances than females (72). Pozo-Cruz et al. (2011) stated that previous studies found similar balance-test results for the dominant and non-dominant leg (73). Also, in the current study, balance of both legs dominant and non-dominant were measured.

Additionally, sense of balance and surefootedness were assessed with two questions: “How do you rate your personal feelings about your balance at this moment?” and “How sure-footed do you feel at this moment?” (17). Answers could range from 0 being “a lot worse than usual”, to 100 being “much better than usual”, and with 50 being “same as always”.

### Cognitive interference measured with the stroop color word test

Cognitive interference was measured with the Stroop Color Word Test (66). In this well-established test (74), participants are given color words that are written in color and are asked to indicate the ink color of the word, thus having to ignore the dominant tendency of reading the word.

In the current study a digital form of the Stroop Color Word Test was applied, using the Inquisit 5 Lab program (Millisecond Software, LLC, Seattle, USA) on a computer. After a test trial, the experimental trial started. It consisted of 84 randomly sampled items with congruent (color word, e.g., “red” and the ink color it is presented in is the same, hence red), incongruent (color word, e.g., “black” and the ink color it is presented in is not the same, e.g., green) and neutral items (colored rectangles in black, red, blue or green). Test duration was approximately 3 min.

Laird et al. (2005) describes cognitive interference to be the difference between incongruent items and a control condition, either congruent, neutral, or non-lexical items (75). Analogous to a previous study on cognitive effects from SR-WBV (76), congruent items are compared to neutral items in the present study. A higher difference between both conditions means higher cognitive interference and thus lower inhibitory control (77).

**Figure 2:**
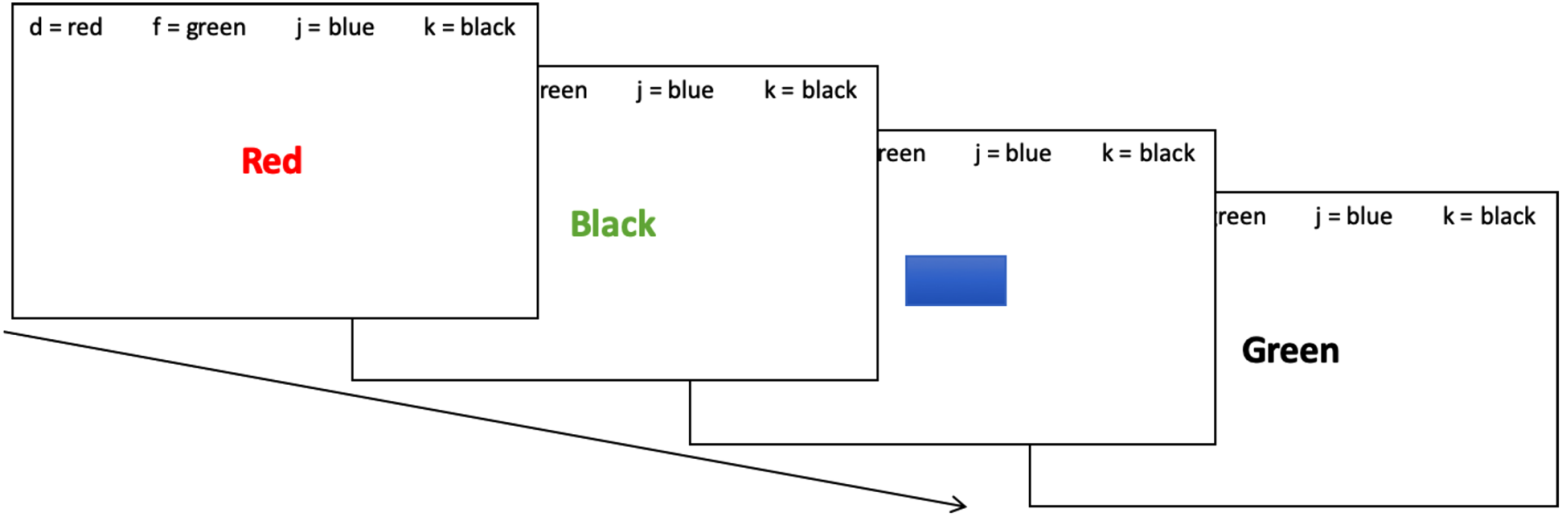
Stroop test on a computer set-up. The Stroop Color Word Test was held on a computer. In a test trial, congruent (color word and the color it is presented in are the same), incongruent (color word and the color it is presented in are not the same) and neutral items (colored rectangles in red, green, blue or black) were shown. After a test trial, the 3-minutes experimental trial with 84 randomly sampled items started.

Because keyboards often differ in latency-time (78), reaction response boxes V1.0 (© immo electronics) were applied instead of keyboards. Four buttons, according to the four presented colors, were placed between participant and computer screen. Participants held index- and middle finger of each hand on the buttons during the test. Figure 2 shows the Stroop Test on a computer set-up.

### Procedure

The experiment was carried out by 2 examiners (MH, SS) in a laboratory room at the University of Bern. While the first examiner guided the participants through the procedure, the second examiner acted as an assistant. These roles were changed regularly to prevent monotony. In order to standardize the procedure, examiners followed a strict case report form (CRF). No more than one participant could attend the experiment, which lasted approximately 50 min.

Before participants arrived, they were randomly allocated to verum (8.5 Hz) or sham (6 Hz) group by flipping a coin. Participants then read through the study-information and signed a consent form to declare their voluntary participation and the possibility to stop the experiment whenever they wanted. Although participants were unaware of their group-allocation or their vibration frequencies, blinding of the examiners was not feasible. The first examiner explained the overall procedure but did not reveal the group allocation. Participants attended the baseline-measurements, starting with the baseline questionnaire, Stroop Color Word Test, flexibility test (mFTF) and balance test (mSEBT).

As in previous studies using an SR-WBV vibrating plate (17, 26), the vibration exercise with the MediPlate® consisted of three series that lasted one-minute each with two one-minute breaks in between. Participants were instructed to stand in the middle of the platform facing forward in an upright position with slightly bent knees (i.e., a skiing posture) and with their arms hanging loosely at their sides. In the short break in between, participants could loosen up and prepare for the following series.

**Figure 3:**
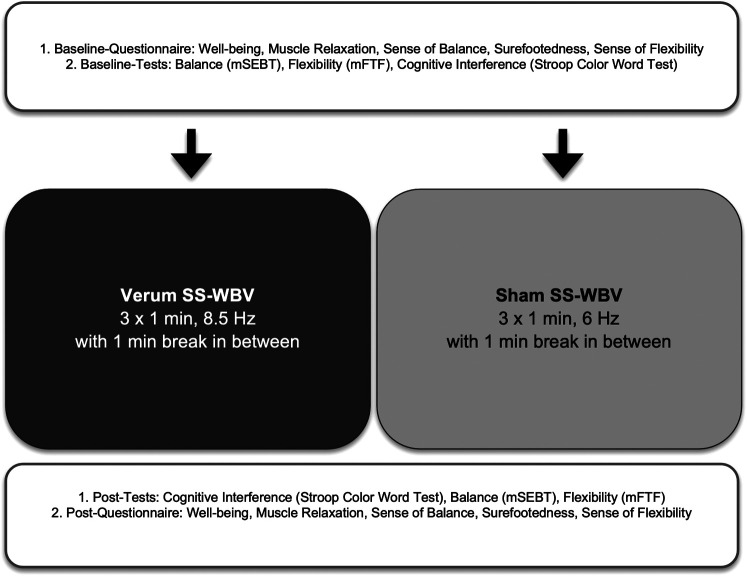
Flowchart of the procedure. Baseline-measurements started with the baseline questionnaire and were followed by the Stroop Color Word Test, flexibility test (mFTF) and then balance test (mSEBT); The sinusoidal side-alternating vibration exercise with the MediPlate® consisted of three series that lasted one-minute each with two one-minute breaks in between; Focusing on immediate effects on cognitive interference, participants attended the post-tests (starting with the Stroop Color Word Test, followed by the mSEBT and then mFTF) before the post-questionnaire.

Focusing on immediate effects on cognitive interference, participants attended the post-measurements in the following order straight after the exercise: Stroop Color Word Test, flexibility test, balance test and questionnaire. The post-questionnaire was longer as it also included demographical questions. The whole procedure is shown in Figure 3.

### Statistical analysis

Musculoskeletal well-being, muscle relaxation, flexibility, balance, and cognitive interference were analyzed in a dependent sample t-test examining differences between baseline and exercising conditions using SPSS (version 25, SPSS, IBM Inc., United States). *P*-values were two-tailed with an *α*-level set at 5%. Collected variables were not approximately normally distributed (*p* < .05) as assessed by Shapiro–Wilk Tests. Thus, graphical approaches, skewness and kurtosis were included in the decision, showing all variables to be close to normal. Also, according to Field (79) analysis of the hypotheses can be considered robust against violations of the normal distribution when the group size is equal. Pearson's descriptive statistics for the collected variables are shown in Table 1. Results of t-tests for each exercising condition are shown in Table 2. Effect sizes are, according to Cohen (80) described as *small* (*d* = 0.2), *medium* (*d* = 0.5), *and large* (*d* ≥ 0.8). The formula for the calculation of effect sizes for dependent *t*-test results is according to Dunlap and colleagues (81):

$$d=t_{c}\sqrt{\frac{2(1-r)}{n}}$$

**Table 1:** Descriptive and inferential statistics.

Variable	Verum SS-WBV 8.5 Hz (*n* = 28)		Sham SS-WBV 6 Hz (*n* = 27)		*t*	*P*
	*M*	SD	*M*	SD		
Sex (m, f)	6m, 22f		5m, 22f		.27	.792
Age (years)	22.36		23.74		−.81	.423
BMI (kg/m²)	21.23		21.58		−.54	.591
Sport (1 “never”−7 “daily+”)	4.79	.92	5.15	1.17	−1.28	.205
Smoking (yes, no)	4y, 24n		3y, 24n			
BL Well-being	75.64	31.84	80.41	18.20	−.68	.50
BL Muscle Relaxation	73.39	30.62	72.56	19.01	.12	.90
BL Balance-Test (dom. leg)	74.81	10.39	72.41	7.28	.99	.327
BL Balance-Test (non-dom. leg)	73.45	7.03	72.82	5.98	.36	.723
BL Sense of Balance	47.50	6.12	48.96	6.50	−.86	.394
BL Surefootedness	48.86	4.20	51.22	7.07	−1.52	.136
BL Flexibility-Test	3.77	9.69	6.25	11.03	−.89	.379
BL Sense of Flexibility	44.04	8.47	48.48	11.85	−1.61	.114
BL Cognitive Interference (RT control)	145.96	136.77	49.24	439.90	−1.05	.298

Sport: Amount of Sport was measured using a 7-point likert-scale from 1 “never” to 7 “several times a day”; being a smoker was answered with yes (y) or no (n); Baseline (BL); BL Variables: Musculoskeletal well-being and muscle relaxation were assessed on a 100-point-rating-scale from 0 “not at all comfortable” to 100 “as comfortable as you can imagine”; Balance was measured with the modified Star Excursion Balance Test (mSEBT); Sense of Balance and Surefootedness were answered from 0 “a lot worse than usual” to 100 “much better than usual” and with 50 being “same as always”; Flexibility was assessed with the modified Fingertip to Floor Test (mFTF); Sense of Flexibility was answered from 0 “not at all flexible” to 100 “as flexible as I can imagine”; Cognitive Interference was calculated as the difference between reaction time (in milliseconds; ms) in incongruent trials minus the reaction time (ms) in control trials. Higher interference stands for lower inhibitory control; *p*-values are two-tailed with an *α*-level set at 5%.

**Table 2:** Results of *t*-tests for each exercising condition.

	Verum SS-WBV 8.5 Hz (*n* = 28)				Sham SS-WBV 6 Hz (*n* = 27)				Intergroup Analysis for *E*	
	BL	*E*			BL	*E*				
	Mean ± SD	Mean ± SD	*t*	*p*	Mean ± SD	Mean ± SD	*t*	*p*	*t*	*p*
Musculoskeletal Well-being	75.64 ± 31.84	88.50 ± 11.54	−2.26	.032	80.41 ± 18.20	76.81 ± 26.15	.93	.359	2.13	.040
Muscle Relaxation	73.39 ± 30.62	85.82 ± 13.53	−2.21	.036	72.56 ± 19.01	76.96 ± 4.85	−1.16	.258	1.63	.109
Balance-Test (dominant leg)	74.81 ± 10.39	82.28 ± 11.76	−5.52	<.001	72.41 ± 7.28	76.90 ± 6.70	−6.01	<.001	2.08	.043
Balance-Test (non-dominant leg)	73.45 ± 7.03	80.05 ± 10.47	−4.52	<.001	72.82 ± 5.98	77.79 ± 7.57	−5.36	<.001	.92	.364
Sense of Balance	47.50 ± 6.12	54.71 ± 10.31	−3.82	.001	48.96 ± 6.50	56.78 ± 10.28	−4.69	<.001	−.74	.461
Surefootedness	48.86 ± 4.20	53.61 ± 9.96	−2.66	.013	51.22 ± 7.07	52.89 ± 8.43	−.80	.429	.29	.774
Flexibility-Test	3.77 ± 9.69	6.21 ± 8.61	−5.63	<.001	6.25 ± 11.03	7.82 ± 11.03	−6.51	<.001	−.60	.548
Sense of Flexibility	44.04 ± 8.47	57.11 ± 12.22	−4.55	<.001	48.48 ± 11.85	56.74 ± 13.40	−3.89	.001	.11	.916
Cognitive Interference (RT control)	145.96 ± 136.77	86.97 ± 101.34	2.14	.042	49.24 ± 439.90	137.36 ± 162.07	−.98	.335	−1.38	.171

Left: Verum sinusoidal side-alternating whole-body vibration (SS-WBV, 8.5 Hz) at Baseline (BL) and exercising condition (*E*); Middle: Sham WBV (6 Hz) at BL and E; Right: Intergroup Analysis for the exercising condition (verum SS-WBV *E* vs. sham SS-WBV *E*); Variables: Musculoskeletal well-being and muscle relaxation were assessed on a 100-point-rating-scale from 0 “not at all comfortable” to 100 “as comfortable as you can imagine”; Balance was measured with the modified Star Excursion Balance Test (mSEBT); Sense of Balance and Surefootedness were answered from 0 “a lot worse than usual” to 100 “much better than usual” and with 50 being “same as always”; Flexibility was assessed with the modified Fingertip to Floor Test (mFTF); Sense of Flexibility was answered from 0 “not at all flexible” to 100 “as flexible as I can imagine”; Cognitive Interference was calculated as the difference between reaction time (in milliseconds; ms) in incongruent trials minus the reaction time (ms) in control trials. Higher interference stands for lower inhibitory control; *p*-values are two-tailed with an *α*-level set at 5%.

## Results

### Participant characteristics

Fifty-five healthy students and acquaintances (44 female; mean age = 23.04 years, *SD *= 6.33 years; mean height = 170.39 cm, *SD* = 9.09; mean weight=63.09, *SD* = 11.29; mean BMI = 21.40, *SD* = 3.03) took part in the current study. All participants were randomly assigned to verum (*N* = 28) or sham (*N* = 27) condition.

Verum and sham groups did not differ significantly in any demographic characteristics or in baseline variables (Table 1).

### Higher musculoskeletal well-being and muscle relaxation after verum SS-WBV (H1)

A significant effect on musculoskeletal well-being was found after verum SS-WBV (*t* = −2.26 *p* = .032*, N* = 28), but not after sham SS-WBV (*t* = 0.93, *p* = .359*, N* = 27). Compared to baseline measurement (verum: 75.64 ± 31.84; sham: 80.41 ± 18.20), musculoskeletal well-being increased significantly after verum (88.50 ± 11.54), but not after sham SS-WBV (76.81 ± 26.15). Effect sizes using Cohen's *d* (75) on musculoskeletal well-being in the verum condition was *d* = −0.495, and in the sham condition *d* = 0.151.

A significant effect on muscle relaxation was found after verum SS-WBV (*t *= −2.21, *p = *0.032, *N* = 28), but not after sham SS-WBV (*t *= −1.16, *p = *.258, *N* = 27). Compared to baseline measurement (verum: 73.39 ± 30.62; sham: 72.56 ± 19.01), muscle relaxation was significantly increased after verum (85.82 ± 13.53), but not after sham SS-WBV (76.96 ± 4.85). Effect sizes using Cohen's d (80) on muscle relaxation in the verum condition was *d* = −0.501, and in the sham condition *d* = −0.192.

### Better balance after verum SS-WBV (H2)

A significant effect in balance (dominant leg) was found after verum SS-WBV (*t *= −5.52, *p *< .001, *N* = 28) and also after sham SS-WBV (*t *= −6.01, *p *< .001, *N* = 27). Compared to baseline measurement (verum: 74.81 ± 10.39; sham: 72.41 ± 7.28), the balance (dominant leg) increased significantly in verum (82.28 ± 11.76) and in sham SS-WBV (76.90 ± 6.70). Effect size using Cohen's *d* (75) on balance (dominant leg) in the verum condition was *d* = −0.663, and in the sham condition *d* = −0.621.

A significant effect in balance (non-dominant leg) was found after verum SS-WBV (*t *= −4.52, *p *< .001, *N* = 28) and also after sham SS-WBV (*t *= −5.36, *p *< .001, *N* = 27). Compared to baseline measurement (verum: 73.45 ± 7.03; sham: 72.82 ± 5.98), balance (non-dominant leg) increased significantly in verum (80.05 ± 10.47) and in sham SS-WBV (77.79 ± 7.57). Effect size using Cohen's d (80) on balance (non-dominant leg) in the verum condition was *d* = −0.689, and in the sham condition *d* = −0.695.

A significant effect in sense of balance was found after verum SS-WBV (*t *= −3.82, *p = *.001, *N* = 28), and also after sham SS-WBV (*t *= −4.69, *p *< .001, *N* = 27). Compared to baseline measurement (verum: 47.50 ± 6.12; sham: 48.96 ± 6.50), sense of balance increased significantly in verum (54.71 ± 10.31) and in sham SS-WBV (56.78 ± 10.82). Effect size using Cohen's *d* (80) on sense of balance in the verum condition was *d* = −0.824, and in the sham condition *d* = −0.86.

A significant effect in surefootedness was found after verum SS-WBV, (*t *= −2.66, *P = *.013, *n* = 28), but not after sham SS-WBV (*t *= −0.80, *p = *.429, *N* = 27). Compared to baseline measurement (verum: 48.86 ± 4.20; sham: 51.22 ± 7.07), surefootedness increased significantly in verum (53.61 ± 9.96), but not in sham SS-WBV (52.89 ± 8.43). Effect size using Cohen's d (80) on surefootedness in the verum condition was *d* = −0.581, and in the sham condition *d* = −0.213.

### Better flexibility after verum SS-WBV (H3)

A significant effect in flexibility was found after verum SS-WBV (*t *= −5.63, *p *< .001, *N* = 28) and also after sham SS-WBV (*t *= −6.51, *p *< .001, *n* = 27). Compared to baseline measurement (verum: 3.77 ± 9.69; sham: 6.25 ± 11.03), flexibility increased significantly in verum (6.21 ± 8.61) and in sham SS-WBV (7.82 ± 11.03). Effect size using Cohen's *d* (80) on flexibility in the verum condition was *d* = −0.261, and in the sham condition *d* = −0.137.

A significant effect in sense of flexibility was found after verum SS-WBV (*t *= −4.55, *p *< .001, *N* = 28) and also after sham SS-WBV (*t *= −3.89, *p = *.001, *N* = 27). Compared to baseline measurement (verum: 44.04 ± 8.47; sham: 48.48 ± 11.85), flexibility increased significantly in verum (57.11 ± 12.22) and in sham SS-WBV (56.74 ± 13.40). Effect size using Cohen's *d* (80) on sense of flexibility in the verum condition was *d* = −1.244, and in the sham condition *d* = −0.650.

### Less cognitive interference after verum SS-WBV (H4)

A significant smaller interference effect was found after verum SS-WBV (*t *= 2.14, *p = *.042, *N* = 28), but not after sham SS-WBV (*t *= −0.98, *p = *.335, *N* = 28). Compared to baseline measurement (verum: 145.96 ± 136.77; sham: 49.24 ± 439.90), the difference in reaction time between incongruent and control items decreased significantly after verum (86.97 ± 101.34), but not after sham SS-WBV (137.36 ± 162.07). Effect sizes using Cohen's *d* (80) on cognitive interference in the verum condition was *d* = 0.486, and in the sham condition *d* = −0.265.

## Discussion

Overall, promising effects were found for the verum WBV condition, but not for the sham condition, indicating acute musculoskeletal and cognitive effects of SS-WBV. More precisely, musculoskeletal well-being and muscle relaxation increased after SS-WBV with a vibration frequency of 8.5 Hz but not of 6 Hz (H1). Also, flexibility assessed through the modified fingertip-to-floor method (mFTF) as well as through a single-item question increased after both conditions, 8.5 Hz as well as 6 Hz. (H2). Sense of balance, which was assessed with a single-item question only increased after SS-WBV with a vibration frequency of 8.5 Hz but not of 6 Hz. However, balance measured with the modified Star Excursion Balance Test (mSEBT) improved after both SS-WBV conditions, 8.5 Hz and 6 Hz (H3). Lastly, cognitive interference measured with the Stroop Color Word Test decreased after SS-WBV with a vibration frequency of 8.5 Hz but not of 6 Hz (H4).

The aim of the current study was to conduct the acute effects of WBV training using a light and portable platform, incorporating the use of several physiological tests and questionnaires. Body balance performance measure included not only musculoskeletal outcomes, but also cognition. After one SS-WBV exercise, different physiological and cognitive measurements have shown improvement, with effect sizes for WBV training being small to moderate. Results might indicate that different variables could be sensitive for different vibration frequencies.

### Whole-body vibration training is beyond physiological effects

WBV training has proven its health promoting effects in various outcomes such as higher musculoskeletal well-being, better flexibility and increased balance (17, 26, 27, 82, 83). Musculoskeletal well-being and flexibility improved after one session of WBV-training only in the verum group. This supports previous findings where it was shown, that WBV increases flexibility (63, 83), because vibration increases blood circulation and generates more heat, which facilitates flexibility. Additionally, WBV causes muscles to contract and relax, which may raise the pain threshold and could lead to participants being able to stretch further while experiencing less pain (84, 85).

Piecha et al. (2014) have shown that WBV increases postural stability (55), which allows us to move safely, which could be related to improved surefootedness. One reason for increased postural stability could be enhanced muscle strength (86). Thus, changes in muscle strength might play a significant role in increasing postural stability and should be addressed in future studies. An increase in surefootedness was significant in the verum group, but not in the sham group, indicating that participants walked more safely after higher vibration stimulation. Balance-Tests however showed not only the verum (8.5 Hz), but also the sham group (6 Hz) increased in balance. One could assume that this result might be related to a training effect on the balance test. However, this might also indicate that WBV also effects balance when light frequencies are applied, maybe because proprioceptive training does not need as high frequencies as e.g., relaxation and musculoskeletal well-being.

Having a good balance is associated with less falls (87), because sensorimotor performance is better (88). But this is not the only explanation. Less falls are also related to better cognitive performance (89, 90), especially with executive functions (91, 92), such as inhibition. For example, Hausdorff et al. (2005) measured inhibition with the Stroop Test in non-demented older adults and have shown that a lower performance in the Stroop Test was also linked to a lower gait performance (93).

Recent studies have shown that cognition may be enhanced through WBV training in mice and in humans (94). In their randomized controlled trial, Boerema and colleagues postulated that daily vibration trainings over 5-weeks improve motor performance and reduce arousal-induced home cage activity in mice (94). In humans, WBV training improved brain function tested with the Stroop Color-Word test. Accordingly, a recently published review from a Brazilian research group on the effects of WBV on different cognitive variables described cognitive enhancement through training and suggests more clinical trials to establish beneficial training parameters (95).

Findings of cognitive effects after WBV training are still rare and the underlying processes are not fully understood yet. Studies with mice have shown increased cholinergic activity after WBV (94). Also, cholinergic activity in humans is positively associated with Stroop Test results (96). Therefore, improvement of inhibitory control in humans may be due to enhanced cholinergic activity increased by WBV training. On the contrary, improved inhibitory control after (repeated) WBV training may be due to the connection of sensory brain regions and the prefrontal cortex. Sensory stimulation, as perceived while executing WBV training, enhances neurotransmission not only in sensory brain regions, but also in the prefrontal cortex (97). However, this finding might be unique for WBV compared to other forms of physical activity (e.g., walking), because Sanders and colleagues have not found any cognitive effects in older persons with dementia after participation in a walking and lower limb strength training program over 12 weeks (98).

Finally, the finding that inhibitory control may be improved through WBV could be an important implication for occupational stress research. Stress at work impairs inhibitory control (99), while inhibitory control is a personal resource that helps to deal with high work demands. Inhibitory control has shown to be related with mindfulness in early adolescence (100) and mindfulness has been shown to be a personal resource that reduces work stress in line with the job demands-resources model (101). According to Lee and Chao (2012), inhibitory control is important for psychological well-being and for achieving mindfulness, and therefore may help to reduce interference from emotional distractors (e.g., an angry face, a negative thought, or a negative event) (102). Thus, people may intentionally avoid emotional distractors and can focus on desired or goal-related information promoting their own well-being (103). This could be noteworthy for future studies exploring personal resources to cope with work demands, but also to reduce cognitions that are related with weaker musculoskeletal function, such as fear- avoidance beliefs, maladaptive back beliefs, and concerns of falling.

The cognitive enhancement was measured with the Stroop Color Word Test. Inhibition and therefore cognition improved from pre to post intervention. Interestingly, these effects were shown in healthy young participants, mostly students who are expected to already have a high level of attention. As in previous WBV-studies on inhibitory control (104–106), a Stroop Test was implemented immediately after the exercise. Future studies may also take long-term effects of SS-WBV on cognition into account.

Further studies may also focus on the aging workforce who could profit the most from SS-WBV interventions focusing on gait performance and frequency of falls, since these have been shown to be related with Stroop Test results (93). In line with this, training parameters concerning different outcome variables must be defined, so users understand which methods (e.g., SR-WBV, SS-WBV), frequencies, and training durations should be applied, if they not only want to increase bone density or reduce musculoskeletal pain, but also improve balance and inhibitory control.

Overall, SS-WBV has shown to be an appropriate way to improve different health-related outcomes. In this initial step, SS-WBV exercise has shown to increase inhibitory control in a young and healthy sample. Implemented as a worksite intervention, SS-WBV is expected to improve balance and reduce falls, especially in older workers.

### Falls and cognitive interference

Research on falls and gait control differ between single falls and recurrent falls. On the one hand, single falls are known as accidental falls, and mostly due to extrinsic reasons, e.g., environmental or housing conditions (88, 89). On the other hand, recurrent falls are often usually based on intrinsic reasons, e.g., advanced age, diseases, or gait disorders (107). Recurrent fallers could profit from interventions such as preventive and therapeutic exercises, in order to improve mobility (108). Because only few effective treatment possibilities exist to effectively improve gait control and balance for fall prevention, new intervention possibilities must be explored (109).

SS-WBV is easily applied and has shown to be effective in improving balance among different studies (26, 63, 64). Interestingly, SS-WBV might affect balance in different ways: Firstly, SS-WBV might improve balance by a proprioceptive training of muscles (110). Secondly, SS-WBV might improve balance through relaxation of stiff muscles, and hence less weakened proprioceptive information in sensory tissues (111, 112), and pain inhibition (113). Thirdly, studies have shown increased inhibitory control, i.e., less cognitive interference, after WBV exercises. This might also indicate a contribution to the prevention of slip trip and fall incidents, because not only gait performance (93), but also falls (90–92) seem to be connected with Stroop Test results. However, underlying mechanisms need to be further explored to fully understand the relationship between WBV, inhibitory control, balance and falls.

### Practical implications

In the current study, WBV exercises were applied on a transportable and manageable vibration platform which does not take up much space and time, because participants are not likely to sweat and would not need to change clothes or shower after WBV-training. Furthermore, WBV-training is very short. In the current study, three minutes of SS-WBV stimulation already showed positive effects. Due to these benefits and the positive physical and cognitive outcomes that were found in the current study, a next step could be to study SS-WBV health-interventions at work. Faes et al. (2018) found promising effects in increased balance and musculoskeletal well-being in office workers who spend a substantial amount of time in sitting position (26). Because several physiological and cognitive measures have been improved, positive effects are not only related to less falls, but may also be linked to better life satisfaction and personal well-being of employees, which could lead to more satisfied employees and better work performances (114, 115).

### Limitations

The described effects of the current study were only observed directly after one training session. Further studies should address long-term effects of repeated SS-WBV trainings. Another limitation of the current study pertains to the “chosen” vibration frequencies. Because the lowest possible frequency (6 Hz) of the SS-WBV platform did not differ enough with the verum condition (8.5 Hz), effects on balance were observed in both conditions. To study effects on balance, the sham-group should possibly experience lower or no vibration frequencies, e.g., control group. However, no vibration frequency would carry the problem of the blindness of participants, because one might guess their group allocation when nothing happens. Finally, our study relates to a relatively young age of participants. Since slip, trip and falls are especially common in the elderly, further studies with older people are necessary.

## Data Availability

The raw data supporting the conclusions of this article will be made available by the authors, without undue reservation.
